# The relationship between apolipoprotein genes polymorphisms and susceptibility to osteonecrosis of the femoral head: a meta-analysis

**DOI:** 10.1186/s12944-018-0827-0

**Published:** 2018-08-17

**Authors:** Yangquan Hao, Hao Guo, Zhaochen Xu, Handeng Qi, Yugui Wang, Chao Lu, Jie Liu, Puwei Yuan

**Affiliations:** 1 Department of Osteonecrosis and Joint Reconstruction, Honghui Hospital Xi’an Jiao Tong University Health Science Center, No. 555 Youyi East Road, Shaanxi 710054 Xi’an, People’s Republic of China; 2 Shaanxi University of Chinese Medicine, Shiji Ave, Xi’an-Xianyang New Ecomic Zone, Shaanxi 712046 Xi’an, People’s Republic of China

**Keywords:** Osteonecrosis of the femoral head, Apolipoprotein A1, Apolipoprotein B, Polymorphism, Genetic model, Meta-analysis

## Abstract

**Background:**

The objective of this study was to evaluate whether apolipoprotein gene polymorphisms confer susceptibility to osteonecrosis of the femoral head (ONFH).

**Methods:**

The relevant literature was screened from databases of Pubmed, Embase, Wanfang, Weipu and China National Knowledge Internet (CNKI) until May, 2017. In addition, odds ratio (OR) and its corresponding 95% confidence interval (CI) were used as a measure of effect size for calculating effect size.

**Results:**

Totally, six case-control studies were included in this meta-analysis. It revealed that *ApoB*-C7623T polymorphism frequency was increased in ONFH group than in control group under three genetic models, including allele model (T vs. C, OR = 4.5149, 95% CI: 1.6968–12.0134); additive model (TC vs. CC, OR = 6.2515, 95% CI: 2.0939–18.6640); and dominant model (TT + TC vs. CC, OR = 5.4998, 95% CI: 1.9246–15.7163). In addition, the increased risk of ONFH were related to *ApoA1*-rs1799837 polymorphism under additive model (AA vs. GG, OR = 1.4175, 95% CI: 1.0522–1.9096) and recessive model (AA vs. GG + AG, OR = 1.7727, 95% CI: 1.3399–2.3452). However, four *ApoB* rs1042031, rs693, 3’-VNTR and G12619A polymorphisms under the all genetic models were not associated with susceptibility to ONFH.

**Conclusion:**

The T allele and TC genotype of *ApoB*-C7623T and AA genotype of *ApoA1*-rs1799837 may contribute to increase the risk of ONFH.

## Background

Osteonecrosis of the femoral head (ONFH) is an intractable disease characterized by the death of osteocytes that caused by obstructed circulation to a specific area, which ultimately leads to inadequate blood supply [1]. Approximately 70–80% ONFH patients can eventually result in collapse of necrotic regions of trabecular bone and cartilage degeneration with altered cartilage function, without timely treatment [2]. The orientation and distribution of collagen fibrils is crucial for the correct distribution of loads in the bone [3]. Vertically oriented collagen fibrils with large diameter are the characteristics in degenerative articular cartilage, and derangement and breakage of collagen network can been observed in the femoral head with collapse [4]. The quality of life of ONFH patients may be seriously affected with increased economic burdens. Reportedly, it is evaluated that about 100,000 to 200,000 new cases are troubled with osteonecrosis annually in China [5]. Although several factors such as alcohol, steroids and hypercoagulability have been demonstrated to be related to the progression of ONFH [6–9], the exact pathologic mechanism of ONFH is still unclear. Therefore, more investigation of the molecular mechanisms of ONFH is needed.

Recently, several studies have demonstrated the genetic polymorphisms of endothelial nitric oxide synthase (*NOS3*) and vascular endothelial growth factor (*VEGF*) contribute to angiogenesis and bone turnover in ONFH patients [10, 11], revealing genetic factors may act as crucial role in the development of ONFH. Apolipoprotein (*APO*), a major blood plasma protein, mediates the transport of lipid by its interaction with cellular receptors [12], and lipid metabolism disorder is considered as one of the leading causes for ONFH development [13]. *APOs* are considered as sensitive markers for estimating lipid metabolic disorder in ONFH populations, whose plasma adiponectin levels are significantly lower than that in healthy controls [14]. Adiponectin level is positively correlated with high-density lipoprotein (HDL), but negatively correlated with triglycerides [14]. Reportedly, the apolipoprotein A1 (*ApoA1*) level is associated with cholesterol homeostasis and lipid metabolism [15]. Similarly, it reveals that the Apo-A1 levels have significant positive correlation with HDL-cholesterol [16]. Notably, the polymorphisms of *APOs* genes may be associated with ONFH with altered lipid metabolism. *ApoA1* rs632153 polymorphism and *ApoB* rs1042034, rs676210, rs673548 polymorphisms are significantly associated with alcohol-induced ONFH in the Han Chinese population [17]. In addition, − 75 G > A polymorphism of *ApoA1* is associated with susceptibility to osteonecrosis in Chinese population [18]. Moreover, apolipoprotein B (*ApoB*) gene polymorphism is relevant with the tendon-vessel stagnation syndrome of steroid-induced ONFH [19].

In the recent years, it has proved that genetic susceptibility is closely relevant with ONFH progression, and SNPs and gene mutation are main factors for genetic susceptibility [20, 21]. Several studies have been performed to explore the associations between *ApoA1* and *ApoB* and the susceptibility to ONFH. However, their results are not consistent and the sample sizes are small. Therefore, we applied this meta-analysis to summarize the interactions between polymorphisms of *ApoA1* and *ApoB* and their susceptibility to ONFH.

## Methods

### Data resources

According to Preferred Reporting Items for Systematic Reviews and Meta-Analyses (PRISMA) guidelines, the present meta-analysis was designed and performed [22]. After predetermining the search strategy, related studies were retrieved by a search of several electronic databases including Pubmed, Embase and Cochrane Library, as well as Chinese electronic databases such as Cochrane Library, Wanfang, Chinese Biomedicine Literature Database (CBM) and China National Knowledge Infrastructure (CNKI) to May 2017, with no language restrictions. The search keywords were “osteonecrosis OR Osteonecroses” OR “femoral head necrosis” OR ONFH OR “Osteonecrosis of the Femeral Head” OR “avascular necrosis of femoral head” OR “necrosis of the femoral head” OR “avascular necrosis of bone” OR “Kienbock disease” OR “Aseptic necrosis of bone” and “APO* OR apolipoprotein OR Apoprotein”.

### Inclusion and exclusion criteria

The studies were needed to adhere to following inclusion criterion: (1) the studies included two groups, ONFH group as experimental group and non-ONFH group as control group; (2) the studies aimed to explore the relationships between the single nucleotide polymorphism (SNP) of *APO* genes and ONFH; (3) the studies could provide the data of genotype or allele frequency of *APO* genes’ polymorphisms in both groups; (4) the article was a case-control study.

In following situations, the study was excluded: (1) the data was incomplete, which could not be applied for statistical analysis; (2) if it was a review, letter or report.

### Data extraction and quality assessment

By using aforementioned methods, two reviewers separately retrieved the databases, as well as extracted the relevant data from included studies. The extracted information included the name of first author, publication time, research region and time, demographic characteristics of cases numbers, age and sex in each group, and the numbers of each genotype of the APO genes’ polymorphism in both groups. Then, the quality of per eligible study was evaluated using Newcastle-Ottawa Scale (NOS) recommended by Agency for Healthcare Research and Quality (AHRQ) [23]. A third assessor was required to resolve the disagreements existed on the course of data extraction and literature quality assessment, until a consensus was reached.

### Statistical analysis

At first, in order to detect the genotype stability of *APO* genes’ polymorphisms in control group, the chi-square test was utilized to perform Hardy-Weinberg equilibrium (HWE) test [24]. Then, the R 3.12 software was used to conduct the present meta-analysis. The odds ratio (OR) and its corresponding 95% confidence interval (CI) were chosen to calculate the pooled effect size of the dichotomous data [25]. The pooled ORs of per included *APO* genes’ polymorphisms under allele model, additive model, recessive model, and dominant model, were respectively calculated to analyze their relationships with the risk of ONFH.

Additionally, heterogeneity among the included studies was detected using chi-square-based Q test and *I*^2^ statistic test [26]. If Q-statistic (*P* < 0.05 or *I*^*2*^ > 50%) was examined, indicating there had significant heterogeneity among those studies, and the pooled result needed to measure under the random-effects model. Otherwise (*P* > 0.05 or *I*^*2*^ < 50%), a fixed-effects model was used [27].

## Results

### Eligible studies and their characteristics

**Fig. 1:**
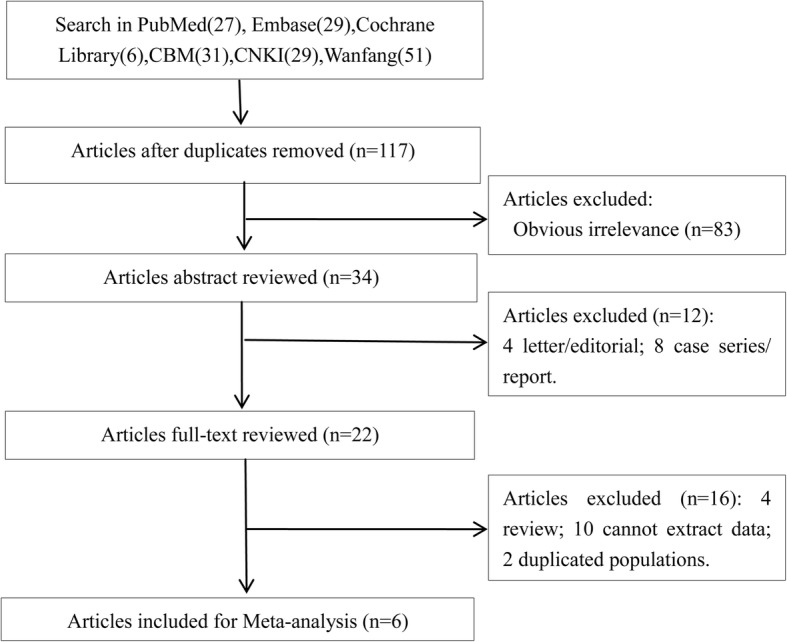
Flow chart shows the selection process of eligible studies

A total of 173 studies were retrieved by using aforementioned strategies: 27 from PubMed, 29 from Embase, 6 from Cochrane Library, 31 from CBM, 29 from CNKI and 51 from Wanfang. After removing 56 duplicates, 117 articles were remained. Then, total 83 articles that did not meet the subject meaning were further excluded by reading the title and abstract of those articles. In addition, 12 articles including 4 letter or editorial and 8 case series or report were also excluded. Afterwards, the remaining 22 articles were full text reviewed, and 16 of them were eliminated due to 4 articles were reviews, 10 with incomplete data and 2 duplicated populations. Therefore, the remaining 6 articles were included in the present study [19, 28–32] (Fig. 1).

A total of 1142 ONFH patients and 920 controls were enrolled in those 6 included studies. The included articles were published during 2007 to 2015, and they were mainly conducted in China and Japan. The NOS scores were ranged from 5 to 7 according to the NOS evaluation system, indicating a high quality. The SNP sites of per included APO genes in present study were *ApoB*-3′-VNTR (variable number of tandem repeats region 3′), *ApoB*-C7623T, *ApoB*-G12619A, *ApoB*-EcoR I (rs1042031), *ApoA1*-G75A (rs1799837) and *ApoB*-Xba I (rs693) (Table 1). The HWE test indicated that the majority of genotypes distribution of controls at above *APO* genes was consistent with HWE (*P* > 0.05), except the *ApoA1*-G75A (rs1799837) in Yin JM et al’ study and Wang XY et al’ studies (*P* < 0.05).

**Table 1:** The characteristics of the included studies

Author, Year, Location	Study Year	NOS	Age	Gender(Male)	SNPs	ONFH				Control				HWE	
			ONFH/Control	ONFH/Control		N	WH	HT	MH	N	WH	HT	MH	Χ^2*^	*P*
Yin JM, [32] 2014, China	2001.11–2013.9	7	44.6 ± 11.3/44.7 ± 11.7	326/278	ApoA1-G75A(rs1799837)	429	215	73	141	368	170	114	84	43.758	< 0.001
					ApoB-EcoR I(rs1042031)	429	391	38	0	368	324	44	0	1.488	0.2226
					ApoB-Xba I(rs693)	429	394	35	0	368	327	41	0	1.281	0.2578
					ApoB-3′-VNTR	429	336	93	0	368	299	69	0	3.938	0.0583
Hirata T, [29] 2007, Japan	1983–2004	7	40.8(20–64)/ 36.1 (9–63)	22/91	ApoB-C7623T	33	27	6	0	122	115	6	1	2.755	0.0969
					ApoB-G12619A	34	33	1	0	123	112	11	0	0.2690	0.6037
					ApoA1-G75A(rs1799837)	33	22	11		120	86	34		–	–
Wang XY, [31] 2008, China	2003.10–2005.9	5	41.6 ± 10.3/40.7 ± 14.7	98/48	ApoA1-G75A(rs1799837)	143	70	20	53	92	45	28	19	10.447	0.0012
					ApoB-EcoR I(rs1042031)	143	125	18	0	92	82	10	0	0.304	0.5815
					ApoB-Xba I(rs693)	143	126	17	0	92	87	5	0	0.072	0.7888
					ApoB-3′-VNTR	142	109	33	0	92	73	18	1	0.009	0.9247
Zeng P, [19] 2014, China	2010.1–2013.12	5	39.85 ± 10.07/41.04 ± 13.61	53/38	ApoB-EcoR I(rs1042031)	108	95	13	0	98	96	2	0	0.010	0.9187
					ApoB-Xba I(rs693)	108	89	19	0	98	92	6	0	0.098	0.7546
Wei XD, [30] 2015, China	2011.1–2012.12	6	39 ± 10/41 ± 14	28/14	ApoA1-G75A(rs1799837)	45	32	13	0	30	28	2	0	0.036	0.8502
					ApoB-Xba I(rs693)	45	41	4	0	30	26	4	0	0.153	0.6956
					ApoB-EcoR I(rs1042031)	45	31	14	0	30	27	3	0	0.083	0.7731
Wei BF, [28] 2011, China	2007.1–2009.12	5	35.17 ± 11.73/34.76 ± 11.94	27/30	ApoB-C7623T	63	57	6	0	71	71	0	0	–	1.000
					ApoB-G12619A	63	56	7	0	71	69	2	0	0.014	0.9042

*SNP* Single Nucleotide Polymorphism, Χ^2*^ *likelihood-ratio Χ*^*2*^, *WH* Wild homozygote, *HT* Heterozygote, *MH* mutational homozygote, *NOS* Newcastle-Ottawa Scale, *N* The total number of including, *ONFH* Osteonecrosis of the Femeral Head, *HWE* Hardy-Weinberg equilibrium tests

### Outcomes

Before quantitative synthesis for per included *APO* genes, an appropriate model was chosen to calculate the pooled effect size. There were striking heterogeneity (*P* < 0.05, *I*^*2*^ ≥ 50%) among the studies associated with *ApoB*-G12619A, rs1042031 and rs693 polymorphisms under all genetic models, as well as rs1799837 polymorphism under allele, recessive and dominant models. Therefore, a random-effects model was used to calculate pooled effect size. Meanwhile, except for those noted above, no remarkable heterogeneity between the studies (*P* > 0.05, *I*^2^ < 50%) related to *ApoB*-rs1799837 on other models, and *ApoB*-3′-VNTR and *ApoB*-C7623T polymorphisms under all genetic models were detected, thus the fix-effects model was used to calculate the pooled OR and 95% CIs.

### Associations between ApoB-3′-VNTR, G12619A, C7623T polymorphisms and ONFH

**Fig. 2:**
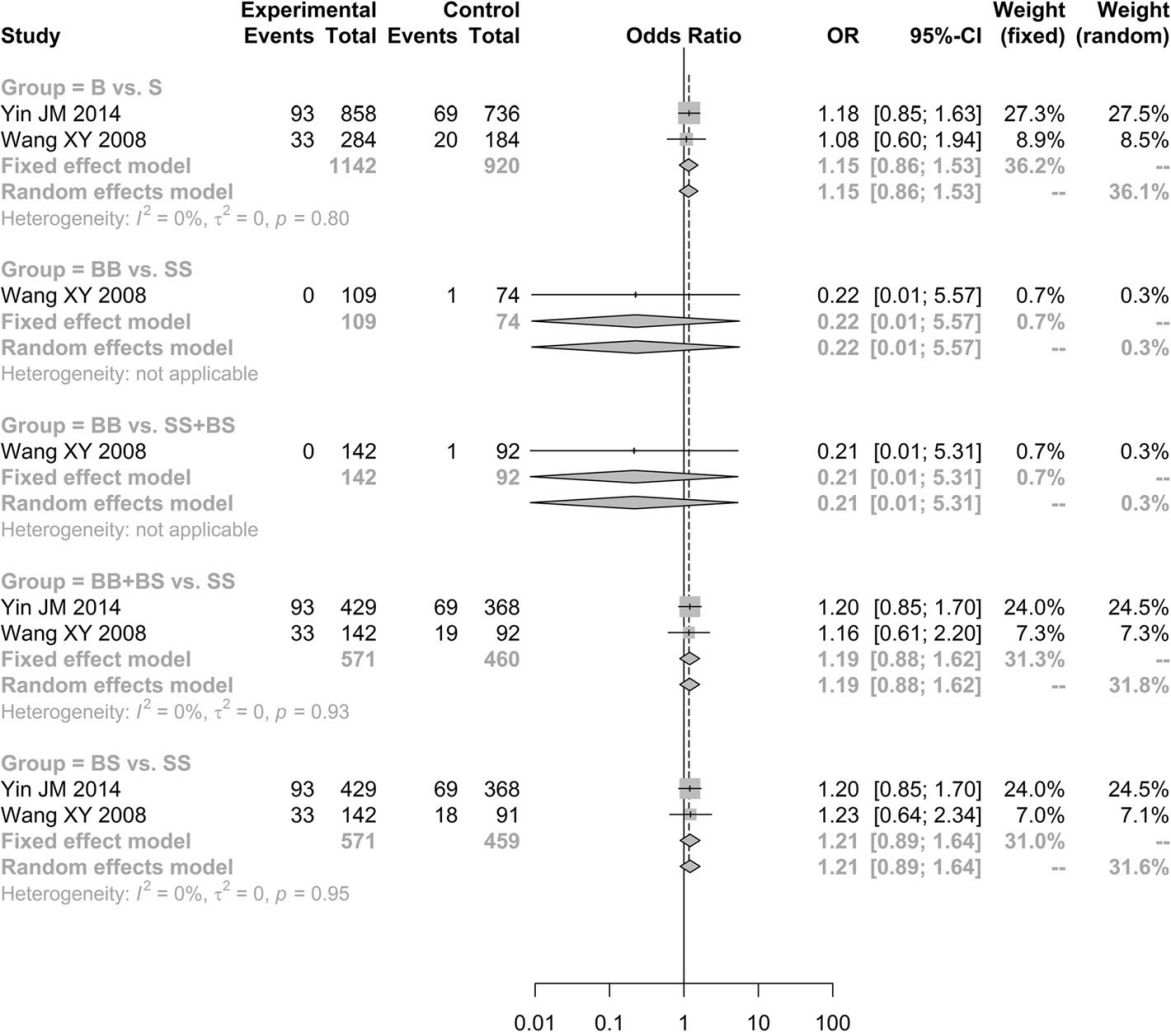
Forest plot of the quantitative synthesis of ApoB-3′-VNTR in ONFH and non-ONFH groups. ONFH: Osteonecrosis of the femoral head. ApoB: apolipoprotein B

Two of six included case-control studies investigated the relationship of *ApoB*-3′-VNTR polymorphism and ONFH risk [31, 32], and another two studies analyzed the *ApoB* C7623T and G12619A polymorphisms [28, 29]. As a result, it showed no significant correlation between the *ApoB*-3′-VNTR polymorphism and ONFH risk under all the genetic models [allele model (B vs. S, OR = 1.1514, 95% CI: 0.8643–1.5339); additive model (BB vs. SS, OR = 0.2237, 95% CI: 0.0090–5.5676); additive model (BS vs. SS, OR = 1.2058, 95% CI: 0.8875–1.6383); recessive model (BB vs. SS + BS, OR = 0.2140, 95% CI: 0.0096–5.3108); dominant model (BB + BS vs. SS, OR = 1.1910, 95% CI: 0.8773–1.6167; Fig. 2)].

**Fig. 3:**
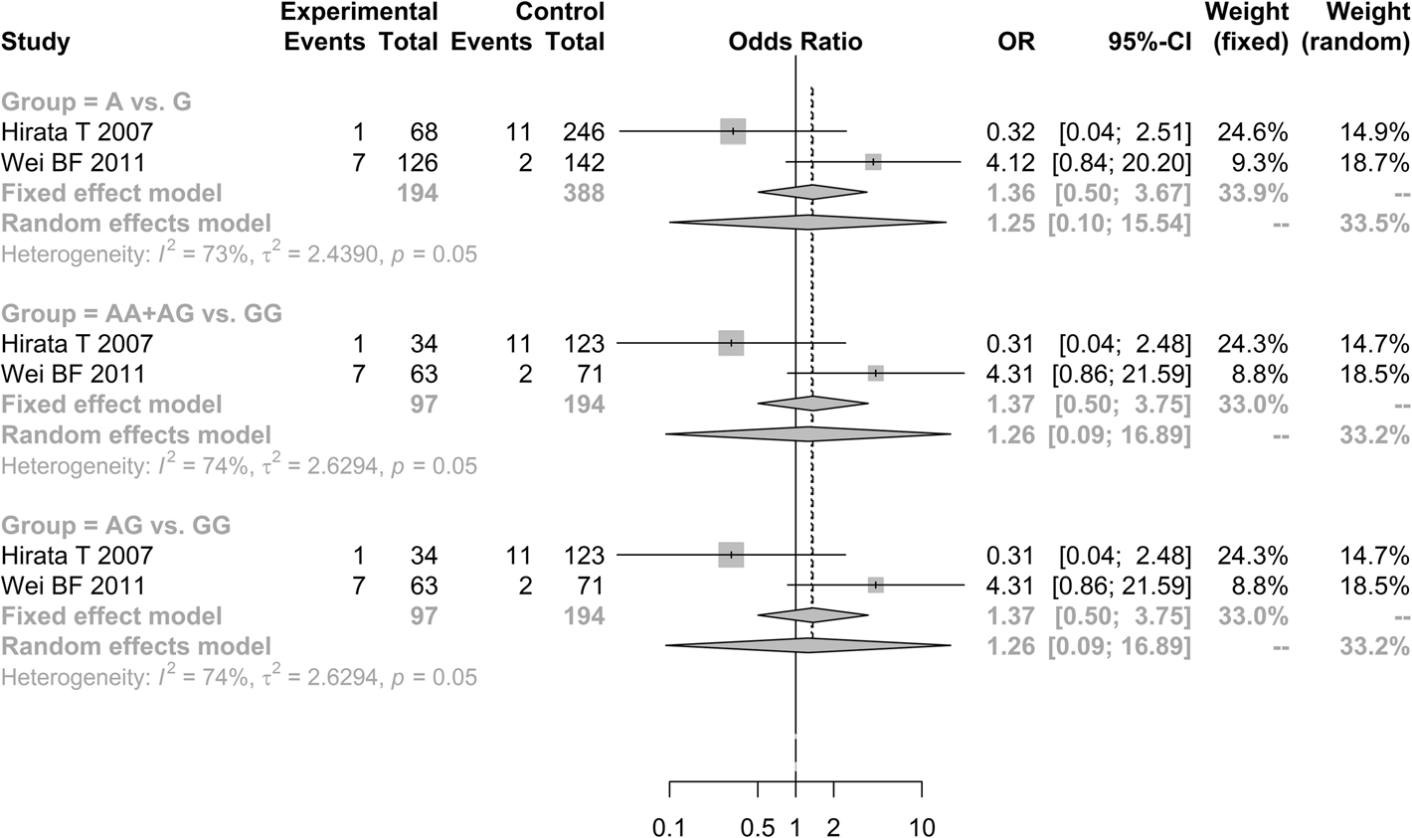
Forest plot of the quantitative synthesis of ApoB-G12619A in ONFH and non-ONFH groups. ONFH: Osteonecrosis of the femoral head. ApoB: apolipoprotein B

Similarly, it also showed lack of significant relationship of *ApoB*-G12619A polymorphism with susceptibility to ONFH under three genetic models [allele model (A vs. G, OR = 1.2499, 95% CI: 0.1005–15.5444); additive model (AG vs. GG, OR = 1.2557, 95% CI: 0.0933–16.8940); dominant model (AA + AG vs. GG, OR = 1.2557, 95% CI: 0.0933–16.8940; Fig. 3)].

**Fig. 4:**
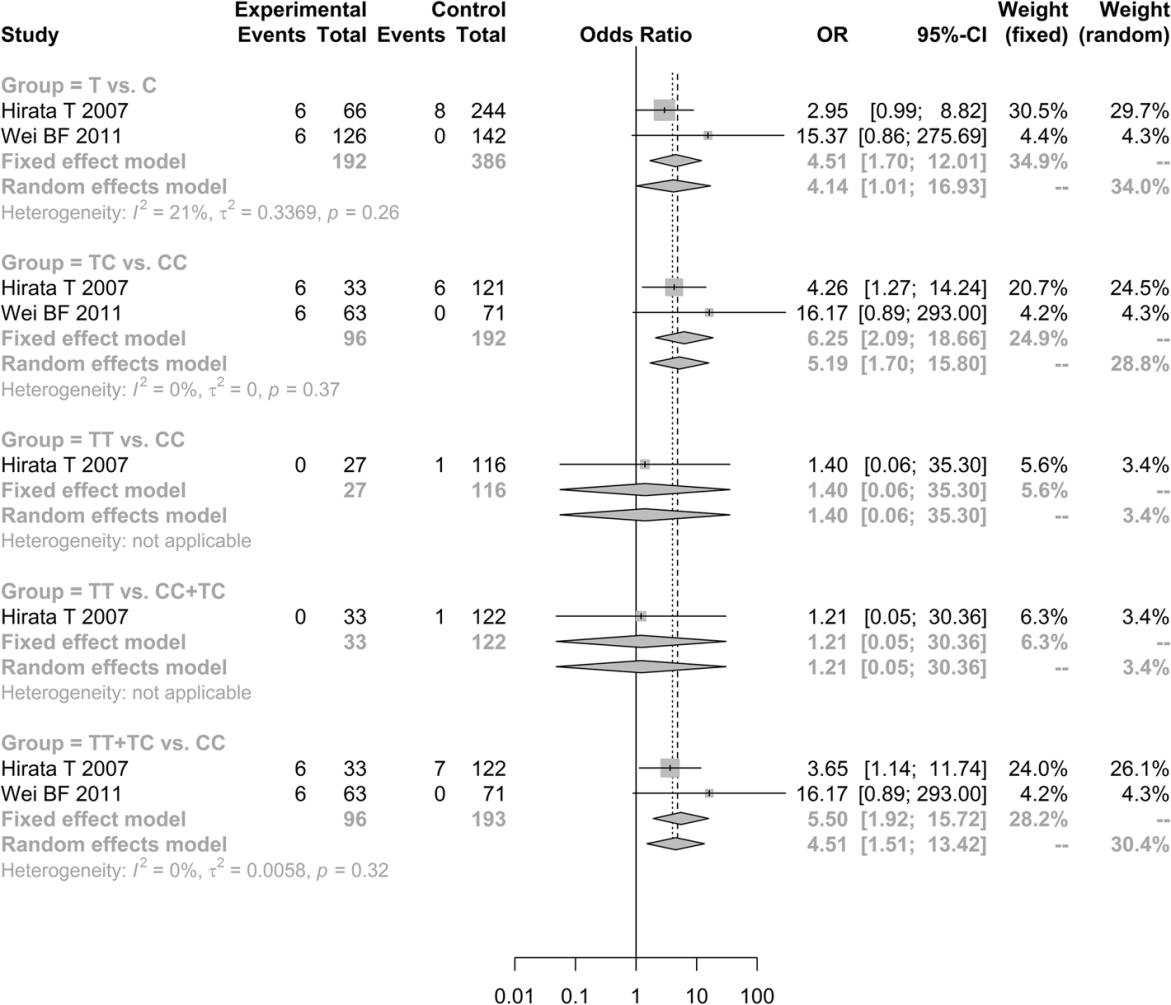
Forest plot of the quantitative synthesis of ApoB-C7623T in ONFH and non-ONFH groups. ONFH: Osteonecrosis of the femoral head. ApoA1: apolipoprotein A1

Notably, the increased *ApoB*-C7623T polymorphism frequencies was observed in ONFH group than in control group under three genetic models [allele model (T vs. C, OR = 4.5149, 95% CI: 1.6968–12.0134); additive model (TC vs. CC, OR = 6.2515, 95% CI: 2.0939–18.6640); and dominant model (TT + TC vs. CC, OR = 5.4998, 95% CI: 1.9246–15.7163; Fig. 4)].

### Associations between ApoB-rs1042031, rs693 polymorphisms and ONFH

**Fig. 5:**
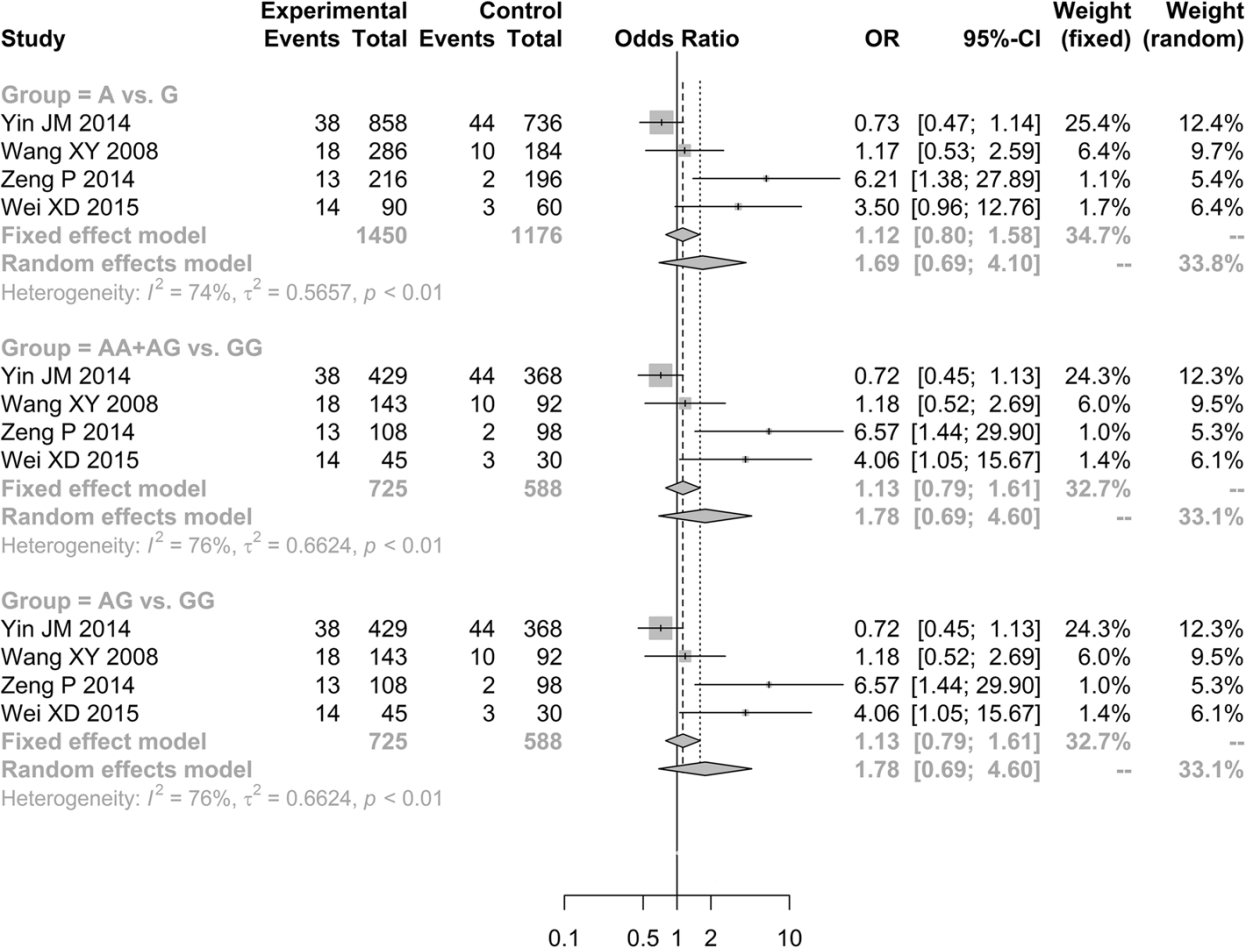
Forest plot of the quantitative synthesis of ApoB-rs1042031 in ONFH and non-ONFH groups. ONFH: Osteonecrosis of the femoral head. ApoB: apolipoprotein B

**Fig. 6:**
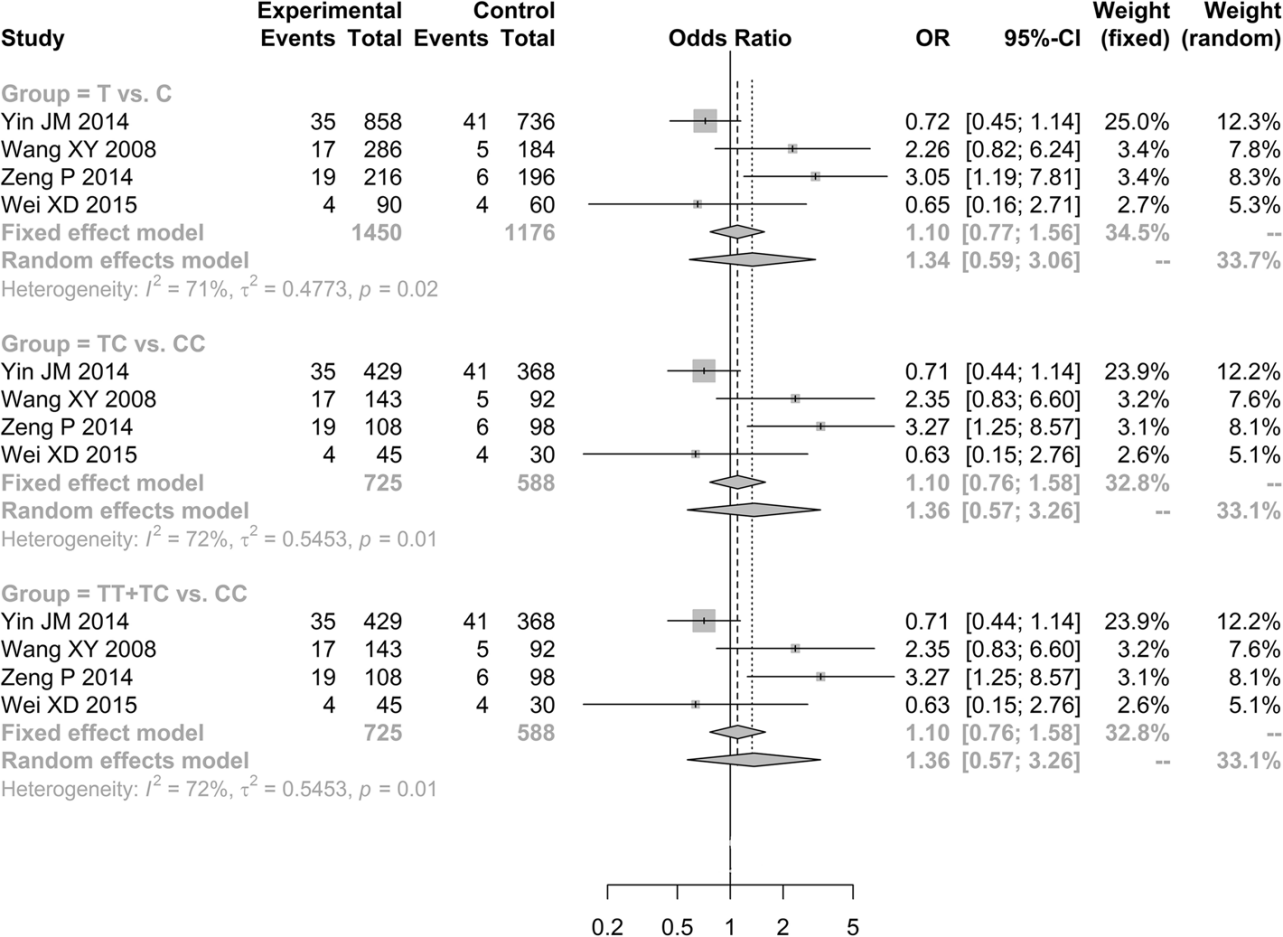
Forest plot of the quantitative synthesis of ApoB-rs693 in ONFH and non-ONFH groups. Squares denote the study-specific outcome estimates, and the size of the square represents the study-specific weight. ONFH: Osteonecrosis of the femoral head. ApoB: apolipoprotein B

Four of the six studies analyzed the rs1042031 and rs693 polymorphisms in *ApoB* gene [19, 30–32]. The outcomes showed that the genetic mutation frequencies of *ApoB*-rs1042031 polymorphism in ONFH patients were not significantly increased than in normal controls under three genetic models [allele model (A vs. G, OR = 1.6863, 95% CI: 0.6928–4.1047); additive model (AG vs. GG, OR = 1.7809, 95% CI: 0.6891–4.6025); dominant model (AA + AG vs. GG, OR = 1.7809, 95% CI: 0.6891–4.6025; Fig. 5)]. In addition, there was no significant difference in rs693 polymorphism between ONFH group and normal group under three genetic models [allele model (T vs. C, OR = 1.3371, 95% CI: 0.5852–3.0553); additive model (TC vs. CC, OR = 1.3630, 95% CI: 0.5695–3.2621); dominant model (TT + TC vs. CC, OR = 1.3630, 95% CI: 0.5695–3.2621; Fig. 6)].

### Association between ApoA1-rs1799837 polymorphism and ONFH

**Fig. 7:**
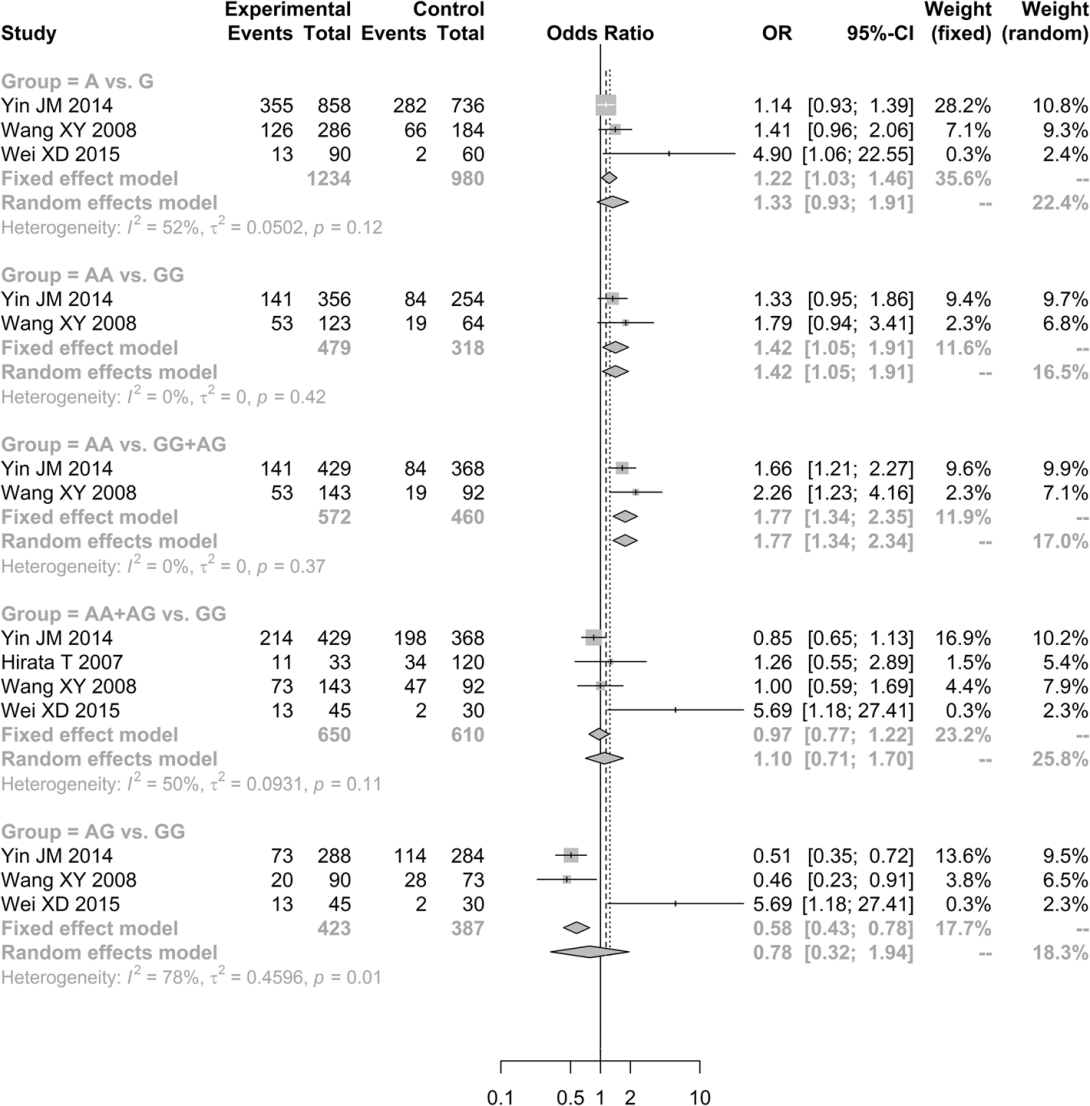
Forest plot of the quantitative synthesis of Apo A1-rs1799837 in ONFH and non-ONFH groups. ONFH: Osteonecrosis of the femoral head. ApoB: apolipoprotein B

A total of four studies assessed the association of *ApoA1*-rs1799837 polymorphism with the occurrence of ONFH [29–32]. The outcomes showed that the *ApoA1*-rs1799837 was associated with the susceptibility to ONFH under additive model (AA vs. GG, OR = 1.4175, 95% CI: 1.0522–1.9096) and recessive model (AA vs. GG + AG, OR = 1.7727, 95% CI: 1.3399–2.3452). However, it showed that the *ApoA1*-rs1799837 polymorphism frequencies in ONFH patients were not significantly increased than in controls under three genetic models [allele model (A vs. G, OR = 1.3317, 95% CI: 0.9275–1.9121); additive model (AG vs. GG, OR = 0.7821, 95% CI: 0.3153–1.9399); and dominant model (AA+AG vs. GG, OR = 1.096, 95% CI: 0.7068–1.6998; Fig. 7)].

## Discussion

In the present study, the relationships of *ApoB*-3′-VNTR, C7623T, G12619A, rs1042031, rs693 polymorphisms and *ApoA1* rs1799837 polymorphism with susceptibility to ONFH were investigated. It showed that *ApoB*-C7623T polymorphism in allele model (T vs. C), additive model (TC vs. CC) and dominant model (TT + TC vs. CC) was closely associated with susceptibility to ONFH. In addition, *ApoA1*-rs1799837 polymorphism was related to the susceptibility of ONFH in additive model (AA vs. GG) and recessive model (AA vs. AG + GG). However, the *ApoB*-3′-VNTR, G12619A, rs1042031 and rs693 polymorphisms were not significantly associated with susceptibility to ONFH.

ApoB and ApoA1 are the major structural and functional protein constituents of the HDL and triglyceride-rich lipoproteins [33]. The apoB plasma level may reflect the concentration of LDL cholesterol [34], while apoA1 protein level is correlated with increased HDL cholesterol [35]. it has been suggested that the increased ratio of low density lipoprotein to high density lipoprotein (LDL/HDL ratio) can result in overloaded fatty emboli, which play an crucial role in the onset of ON [36, 37]. Additionally, it is considered that the elevated level of apoB/apoA1 ratio acts as a potential risk for ON [38]. Those abnormal lipid metabolisms may increase the risk of ONFH via impacting bone repair and reconstruction [39, 40].

In the present study, the frequencies of T allele and TC genotype at *ApoB*-C7623T locus in ONFH patients were significantly higher than those in non-ONFH patients or controls. In addition, the additive model (AA vs. GG) and recessive model (AA vs. AG + GG) in *ApoA1*-rs1799837 were also significantly associated with the increased risk of ONFH. Similarly, two studies have found that the TT or TC genotypes of *ApoB*-C7623T and A allele of *ApoA1*(rs1799837) are significantly correlative with higher risks of ONFH [28, 29]. Additionally, Yin et al. have demonstrated that *ApoA1*-75 bp G > A (rs1799837) is associated with the levels of total cholesterol and HDL in the serum lipid levels [41, 42]. In addition, Hirata et al. have found that there is no significant relationship of the GA or AA genotypes of *ApoA1*-rs1799837 with susceptibility to ONFH in Japanese population [29]. Conversely, the GA or AA genotypes frequency of *ApoA1*-rs1799837 are remarkably higher in the ONFH patients than in normal controls from Chinese population [30]. Thus, the racial differences might be the cause of the difference.

Moreover, although it revealed the four *ApoB* rs1042031, rs693, 3’-VNTR and G12619A polymorphism under all genetic models were not strongly linked to the high risk of ONFH in our work, the pooled OR of those four *ApoB* polymorphisms were higher in ONFH group. The AG and AA genotypes frequency of *ApoB*-rs1042031 are higher in the dyslipidemia group [43]. Meanwhile, the rs1042031 and rs693 polymorphisms of *ApoB* are the lipid profile genetic related in the Kuwait population [44].

In spite of some important findings are revealed in the present study, several limitations are still existed. Firstly, there was significant heterogeneity between the included studies, which might be caused by the different research regions. Meanwhile, the gender and age might also account for the high heterogeneity. Phillips et al. have suggested that gender regulates the association between *ApoB* and *ApoA1* gene polymorphisms and metabolic syndrome risk [33]. Secondly, covariate adjustment and subgroup analysis were not performed, and it might potentially influence the results of the present study. Thirdly, the findings might only be appropriate for the China and Japanese populations. Fourthly, the genotype frequency of Apo AI-G75A (rs1799837) for control group Yin JM et al’ study and Wang XY et al’ studies were not consistent with HWE, indicating that the selected samples in control group might be unable to represent the population in the area. Fifthly, publication bias wasn’t evaluated since the number of included studies < 10 was limited to conduct Egger’s test.

## Conclusion

To summarize, the present study suggested that the T allele and TC genotype of *ApoB*-C7623T, *ApoA1*-rs1799837 under allele, additive and dominant models might contribute to increase the risk of ONFH in both China and Japanese populations. In addition, it revealed that the four *ApoB* rs1042031, rs693, 3’-VNTR and G12619A polymorphisms under all genetic models were not strongly associated with the increased risk of ONFH. However, larger scale case-control studies with more patients and controls participants should be included to further assess the interaction of above *ApoB* and *ApoA1* polymorphisms with ONFH susceptibility.

## Abbreviations

AHRQ: Agency for Healthcare Research and Quality
*APO*: Apolipoprotein
*ApoA1*: Apolipoprotein A1
*ApoB*: Apolipoprotein B
CBM: Chinese Biomedicine Literature Database
CI: Confidence interval
HWE: Hardy-Weinberg equilibrium
NOS: Newcastle-Ottawa Scale
*NOS3*: Nitric oxide synthase
ONFH: Osteonecrosis of the femoral head
OR: Odds ratio
PRISMA: Preferred Reporting Items for Systematic Reviews and Meta-Analyses
SNP: Single nucleotide polymorphism
*VEGF*: Vascular endothelial growth factor
